# Persistent and robust antibody responses to ChAdOx1-S Oxford-AstraZeneca (ChAdOx1-S, Covishield) SARS-CoV-2 vaccine observed in Ugandans across varied baseline immune profiles

**DOI:** 10.1371/journal.pone.0303113

**Published:** 2024-07-29

**Authors:** Jennifer Serwanga, Gerald Kevin Oluka, Claire Baine, Violet Ankunda, Jackson Sembera, Laban Kato, Joseph Ssebwana Katende, Geoffrey Odoch, Betty Oliver Auma, Ben Gombe, The COVID-19 Immunoprofiling Team, Monica Musenero, Pontiano Kaleebu

**Affiliations:** 1 Viral Pathogens Theme, MRC/UVRI & London School of Hygiene and Tropical Medicine, Uganda Research Unit, Entebbe, Uganda; 2 Department of Immunology, Uganda Virus Research Institute, Entebbe, Uganda; 3 Science, Technology, and Innovation Secretariat, Office of the President, Government of Uganda, Kampala, Uganda; Sheikh Hasina National Institute of Burn & Plastic Surgery, BANGLADESH

## Abstract

Understanding SARS-CoV-2 vaccine-induced antibody responses in varied antigenic and serological prior exposures can guide optimal vaccination strategies for enhanced immunogenicity. We evaluated spike (S)-directed IgG, IgM, and IgA antibody optical densities (ODs) and concentrations to the two-dose ChAdOx1-S Oxford-AstraZeneca (ChAdOx1-S, Covishield) SARS-CoV-2 vaccine in 67 Ugandans, categorised by prior infection and baseline S-IgG histories: uninfected and S-IgG-negative (n = 12); previously infected yet S-IgG-negative (n = 17); and previously infected with S-IgG-positive status (n = 38). Antibody dynamics were compared across eight timepoints from baseline till nine months. S-IgG antibodies remained consistently potent across all groups. Individuals with prior infections maintained robust S-IgG levels, underscoring the endurance of hybrid immunity. In contrast, those without prior exposure experienced an initial surge in S-IgG after the primary dose but no subsequent significant increase post-boost. However, they reached levels parallel to the previously exposed groups. S-IgM levels remained moderate, while S-IgA persisted in individuals with prior antigen exposure. ChAdOx1-S, Covishield vaccine elicited robust and sustained antibody responses in recipients, irrespective of their initial immune profiles. Hybrid immunity showed higher responses, aligning with global observations. Early post-vaccination antibody levels could predict long-term immunity, particularly in individuals without virus exposure. These findings can inform vaccine strategies and pandemic management.

## Introduction

As of July 30, 2023, the global count of COVID-19 cases had surpassed 768 million, resulting in more than 6.9 million fatalities; (WHO, 2023; http://covid19.who.int) but, diminished testing and reporting have potentially underestimated infection prevalence. The COVID-19 pandemic, caused by the severe acute respiratory syndrome coronavirus 2 (SARS-CoV-2), significantly impacted nations worldwide, including sub-Saharan Africa (SSA). However, SSA had a comparatively milder impact of the disease, possibly due to its youthful population, potential cross-protection from endemic illnesses, distinctive demographic and environmental factors, and limited international travel [1]. Despite global efforts to combat the virus, SSA faced unique economic challenges and social disparities, necessitating equitable vaccine access. The COVID-19 Vaccine Global Access (COVAX) initiative played a critical role in ensuring fair distribution of the vaccines to low-income nations [2], with ChAdOx1-S, Covishield emerging as the first to be administered in the region. Introducing ChAdOx1-S, Covishield to a mixed baseline exposure SSA population calls for better understanding of its immunogenicity in that setting to bridge the crucial gap in global data. Various global research, including the USA, Chile, Peru, and the UK, showed the vaccine’s capability to consistently elicit robust antibodies, which peaked 28 days post-prime across all age brackets [3]. However, SSA studies on antibody responses to vaccination with ChAdOx1-S, Covishield are limited [4, 5], yet important for understanding immune responses across diverse populations, to inform effective vaccination strategies. Available studies mostly focused on seroprevalence, concurrent cross-reactivity and prior exposure to other human coronaviruses [6, 7], offering only a partial picture of vaccine-induced immunity. Inconsistencies have occurred across ChAdOx1-S, Covishield studies from UK and South Africa [8], possibly due to variable immune response to different SARS-CoV-2 variants [9, 10].

The diverse genetic backgrounds in SSA, influenced by inter-race variations in human leukocyte antigen (HLA) molecules [11], may also contribute to different SARS-CoV-2 immune responses. Others have shown that unique genetic differences in Sub-Saharan Africa can differentially affect vaccine-elicited responses [12–14]. An active immune microenvironment in African individuals has been shown to hinder the effectiveness of vaccinations [15]. A recent study by Metcalf and colleagues revealed that Schistosoma mansoni infection prior to hepatitis B vaccination led to significant alterations in the host’s pre-vaccination environment that resulted in attenuated immune responses to the hepatitis B vaccine [16]. Comparative analysis demonstrated significantly higher antibody titers in convalescent South Africans than those without prior infection, regardless of the studied variant [17]. Immunogenicity of the ChAdOx1-S, Covishield vaccine was demonstrated in two West African cohorts; however, diminishing antibody levels after the second dose implied the necessity for boosting, even in the presence of hybrid immunity resulting from prior infection [18]. Available studies in SSA were conducted on previously exposed individuals, limiting their generalizability. Understanding the immunological performance of these vaccines in SSA populations with varying exposure contexts is critical in informing the region’s vaccination policies. Therefore, the comprehensive assessment of immune responses to ChAdOx1-S, Covishield vaccine in a SSA genetic and environmental context is necessary to inform vaccination strategies for the region. To bridge this gap, we analysed how SARS-CoV-2 -directed IgG, IgM, IgA antibodies evolved and persisted in ChAdOx1-S, Covishield-vaccinated Ugandans, with or without pre-existing anti-spike antibodies over nine months.

## Materials and methods

### Study population

To accomplish the study objectives, we assembled a participant cohort from two preexisting cohort groups. The first group contained 86 convalescent individuals whose prior primary infection was confirmed through RT-PCR and had encountered either very mild or asymptomatic COVID-19 illness. These were recruited at the start of the epidemic through purposive sampling as part of the national guidelines at that time to isolate all individuals who tested PCR positive for COVID-19. Their median age was 29, with an interquartile range (IQR) of 24.0 to 37.5 years. Of these, 18.6% were females, 73.3% were males, and 8.1% lacked gender records (Table 1). Regarding primary symptoms, 15.1% manifested mild symptoms in their previous infection, 39.5% remained asymptomatic, and 45.3% were unclassified because they lacked symptom records. We analyzed 382 samples collected from 86 individuals with prior SARS-CoV-2 infection, spanning the period from February 24, 2021, to August 3, 2022. Based on previous computations in this population [7], a less than two-fold rise in N-IgG antibody levels during follow-up was assumed to indicate absence of re-infection, and antibody naïve individuals were those with no detectable anti-spike antibodies at baseline.

**Table 1:** Participant demographics.

Characteristic	Total Participants (n = 67)	Inf- and S-IgG- (n = 12)	Inf+ and S-IgG- (n = 17)	Inf+ and S-IgG+ (n = 38)
Age, Mdn (IQR)	29.9 (24.8–38.0)	29.7 (28.1–50.5)	32.0 (27.5–35.5)	29.0 (23.5–37.5)
Gender, n(%)				
Female	18 (26.9%)	6 (50%)	1 (5.9%)	11 (28.9%)
Male	46 (68.7%)	6 (50%)	14 (82.4%)	26 (68.4%)
Unknown^#^	3 (4.5%)		2 (11.8%)	1 (2.6%)

^#^Gender was not captured in 3 participants

This table presents the characteristics of the overall study population, which consisted of 67 individuals. These individuals were further categorized based on their infection and serological status into three groups: Inf- and S-IgG- (n = 12) as individuals who tested negative for infection and were also negative for the presence of specific IgG antibodies against the pathogen; Inf+ and S-IgG- (n = 17) as individuals who tested positive for infection but were negative for specific IgG antibodies against the pathogen, and Inf+ and S-IgG+ (n = 38) as Individuals who tested positive for infection and were also positive for specific IgG antibodies against the pathogen.

The second group included 20 presumed naïve subjects, with median age of 30.7 years (IQR: 28.7–40.6), followed between March 9, 2021, and January 28, 2022, to collect 136 samples. These comprised 50% females, 45% males, and 5% with unrecorded gender data. Of these, seven were S-IgG antibody seropositive at baseline, suggesting the possibility of prior exposure, and one lacked the baseline sample. Consequently, the seven subjects displaying baseline S-IgG+ status in this group were reclassified to have had prior infections.

The combined cohort of 106 participants (86 prior infected and 20 presumed infection-naïve) was then stratified into three subgroups, based on their baseline S-IgG seropositivity and prior infection status. The first subgroup, "No prior Infection and Baseline S-IgG Negative” labeled "Inf- and S-IgG-," included 12 subjects with a median age of 29.7 years (IQR: 28.12–50.48). Gender distribution was evenly distributed, with 50% females and 50% males. They provided 91 samples collected between March 11, 2021, and January 10, 2022. The second subgroup, "Prior Infection and Baseline S-IgG Negative (Inf+ and S-IgG-)," contained 17 previously infected participants. These contributed 91 samples between February 25, 2021, and March 15, 2022. Their median age was 32 years (IQR: 27.5–35.5), containing one female, 14 males, and two who lacked gender records. Of these, three were asymptomatic, and 14 displayed no documented admission symptoms. The third subset, termed "Prior Infection and Baseline S-IgG Positive (Inf+ and S-IgG+)," included 38 participants, with 11 females (28.9%), 26 males (68.4%), and one (2.6%) with unrecorded gender. Their median age was 29 years (IQR: 23.5–37.5). Between February 24, 2021, and March 2, 2022, a total of 38 individuals, comprising 13 asymptomatic, four mildly symptomatic, and 21 with no documented admission symptoms, were monitored, and 224 samples were collected. The remaining individuals could not be categorised into the above-mentioned subgroups due to the absence of baseline samples. Thus, our subsequent analysis investigates 67 subjects categorised as 12 "No prior Infection and baseline S-IgG Negative," 17 "Prior Infection and baseline S-IgG Negative," and 38 "Prior Infection and baseline S-IgG Positive," traversing the period from March 11, 2021, to March 15, 2022. All 67 participants received the ChAdOx1-S, Covishield vaccine, and these categories form the basis for our subsequent in-depth analyses and overall conclusions, delineating the vaccine-elicited antibody response throughout nine months. Ethical approval was obtained from the Uganda Virus Research Institute’s Research and Ethics Committee (GC/127/833) and the Uganda National Council for Science and Technology (HS637ES). Written informed consent was obtained from all study participants.

### Study design

**Fig 1:**
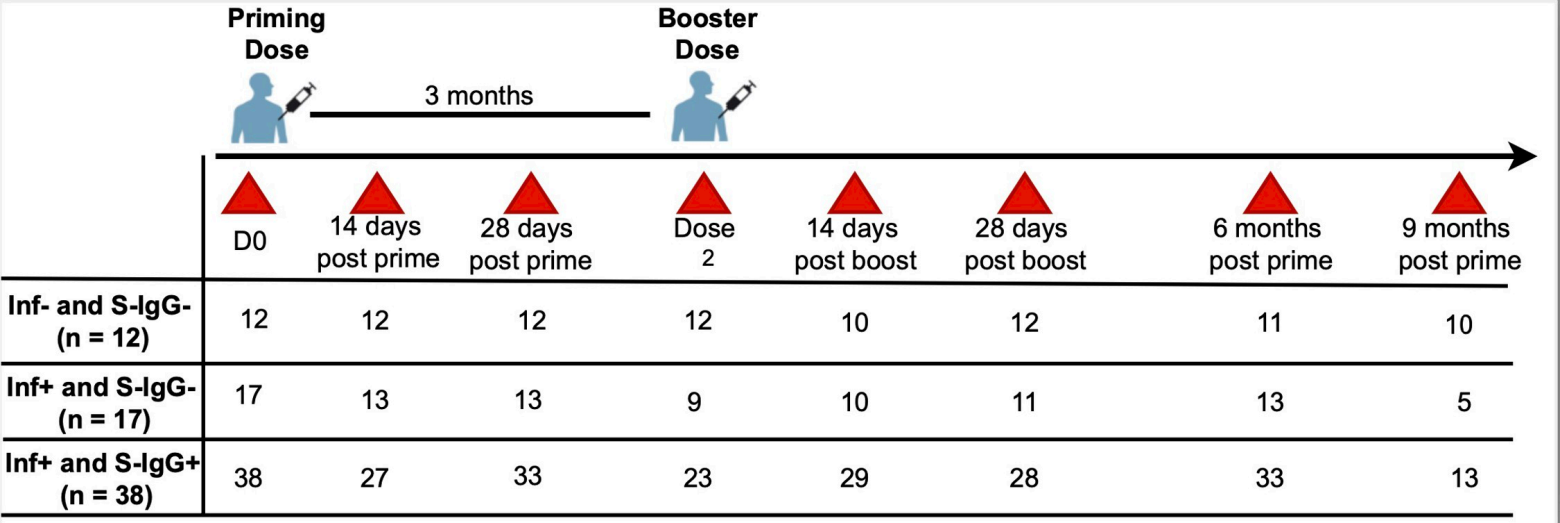
Vaccination and specimen collection schedule. **Fig 1:** depicts the schedule for vaccination and sampling, detailing the quantity of samples collected at each time point for each respective group.

Blood samples were collected before or within a week of administering the first of the two-dose ChAdOx1-S, Covishield regimen, followed by additional samples on Days 14 and 28 after the initial dose. For the booster dose, samples were collected around 90 days after the initial vaccination, with further samples taken on Days 14 and 28 post-booster and at the six- and nine-month mark following the initial dose, summarised in Fig 1.

### Conventional in-house ELISA to detect virus-specific IgG, IgM, and IgA antibodies

We used a previously validated in-house ELISA [19] in which duplicate plate wells per sample were coated with antigen concentration of 3ug/ml, resulting in 0.15ug of antigen per well, to measure SARS-CoV-2-specific IgG, IgM, and IgA antibodies against the spike and nucleoproteins (S and N), described in detail before. Cut-off values for anti-spike antibody seropositivity were 0.432, 0.459, and 0.226, while those for the nucleoprotein were 0.454, 0.229, and 0.225, previously determined. Quality checks included predetermined negative and positive plasmas, CR3009 (2 mg/ml) for N-, CR3022 (0.1 mg/ml) monoclonal antibodies for S-directed antibodies as controls, and duplicate blank wells. The OD values at 450 nm were measured with a BioTek GEN5 software and an ELx808 microplate reader. Blank well values subtracted to obtain the net OD responses.

### Assessing the binding concentrations of IgG, IgM, and IgA antibodies

Vaccine-induced antibody concentrations were quantified using the validated indirect ELISA method mentioned above [19]. Briefly, serially diluted human IgG, IgM, and IgA antibody standards (Sigma #12511, #18260, and #12636) were immobilised onto anti-human kappa and lambda capture antibodies from Southern Biotech (#2060–01, #2070–0). The OD450 values of the standards were used to generate 4-parameter logistic standard curves using the BioTek GEN5 software, allowing estimation of antibody concentrations within the linear range of the curves after adjustment for dilution factors. Antibody concentrations below the detection limit were recorded as 0 ng/ml.

### Statistical analysis

Continuous variables were summarised using descriptive statistics, while categorical variables were presented as frequencies. Individual profile plots showed the temporal evolution of the antibody OD values (nm) and concentrations (ng/ml). Boxplots were used for OD value and concentration comparisons, with the Wilcoxon rank sum test assessing differences among the three subgroups at each time point. A Bonferroni correction was used to adjust for multiple testing.

## Results

### Differential seroconversion dynamics of S-IgG, S-IgM, and S-IgA antibodies after vaccination of subjects with prior infection and baseline S-IgG positivity showing robust S-IgG maintenance, S-IgM decline, and S-IgA persistence

**Fig 2:**
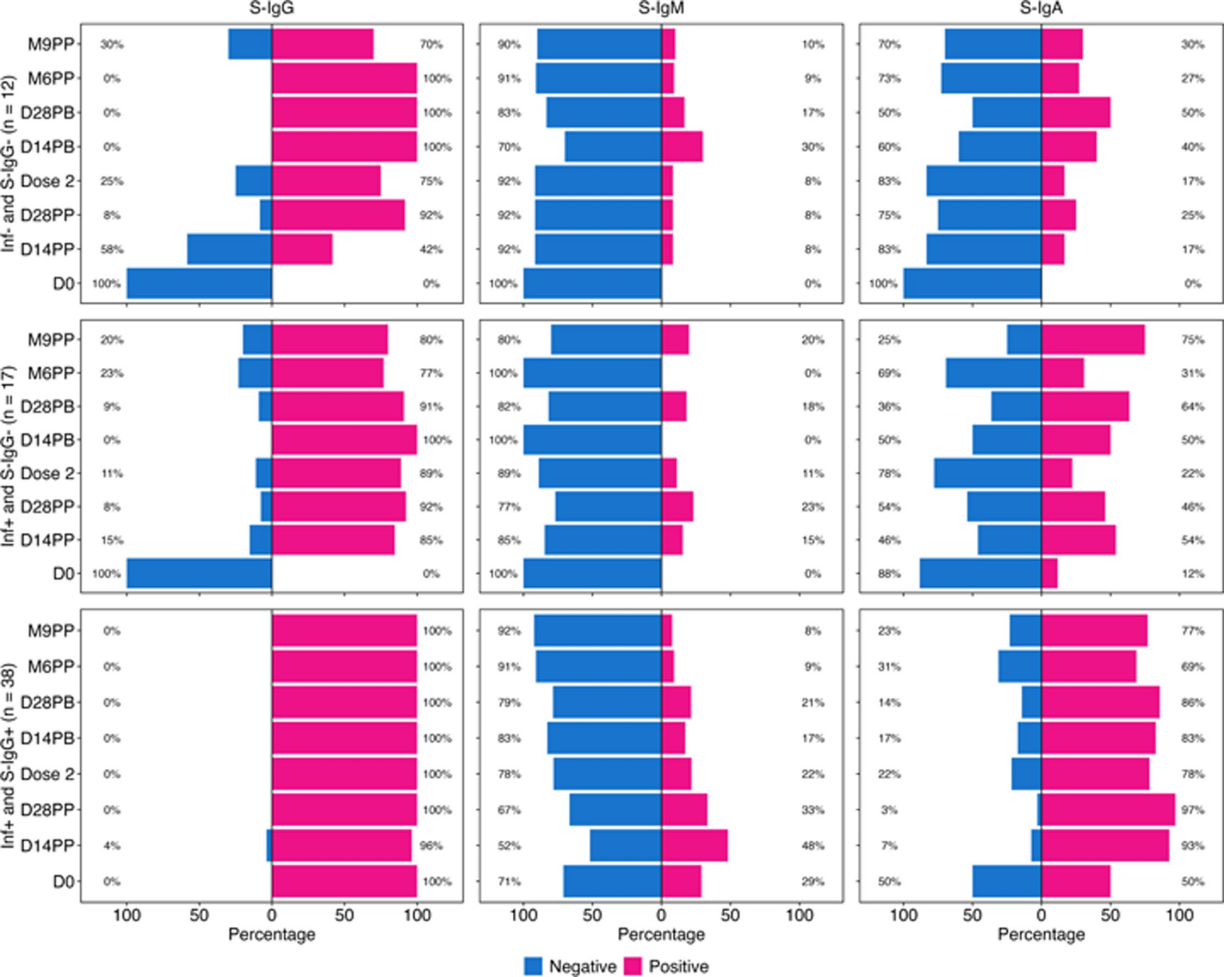
Seroconversion percentages at each time point. **Fig 2** presents the distribution of seroconversion percentages among three distinct subject subgroups: Inf- and S-IgG- (denoted as 2A, 2B, 2C), Inf+ and S-IgG- (denoted as 2D, 2E, 2F), and Inf+ and S-IgG+ (denoted as 2G, 2H, 2I). This distribution is further stratified based on the presence of S-IgG, S-IgM, and S-IgA antibodies, highlighting responders categorised by their positivity (pink) or negativity (blue) at each specimen time point.

The analysis showed that among 17 participants who were originally PCR-positive but later tested negative for SARS-CoV-2 IgG antibodies, the median time between PCR-positive diagnosis and subsequent negative serological screening was 9.03 months. This means that, on average, individuals in this group needed around nine months to clear detectable levels of SARS-CoV-2 IgG antibodies following their initial PCR-positive diagnosis. The median duration of approximately nine months suggests a reasonable timeframe for the decline of detectable S-IgG antibodies to baseline levels prior to vaccination. We first assessed the seroconversion dynamics in individuals lacking prior infection and seronegative for S-IgG, S-IgM, and S-IgA at baseline. At baseline, on day 0 (D0), all participants lacked detectable S-IgG antibody responses. After administration of the first dose, S-IgG seropositivity surged to 42% within two weeks, reaching full seropositivity by 14 days post-boost and sustaining this high level until 9 months. A subset of 30% of the 12 baseline seronegative subjects showed waning of the S-IgG seropositivity by month 9 (Fig 2A). The frequency of S-IgM responders was low, peaking to 30% at two weeks post-boost (Fig 2B). Interestingly, S-IgA responses gradually rose, reaching 50% by 28 days after boosting (Fig 2C). These data highlight the progressive seroconversion patterns of S-IgG, S-IgM, and S-IgA antibodies following vaccination in the absence of a prior infection.

In previously infected, baseline S-IgG naïve subjects, all were S-IgG negative at baseline (D0), but S-IgG seropositivity rapidly rose to 85% by 14 days after the first dose. Subsequent administration of the booster dose resulted in complete seroconversion, reaching 100% positivity by day 14 followed by a declining trend (Fig 2D). The S-IgM response in this cohort remained notably low, settling around 20% over the study duration (Fig 2E). On the contrary, S-IgA-seropositivity increased from an initial baseline of 12% to 54% by 14 days post-prime (Fig 2F). The kinetics of antibody development in individuals with prior infections shed light on the robustness and persistence of hybrid S-IgG, S-IgM, and S-IgA in this group.

Participants with prior infection and initial S-IgG seropositivity consistently had elevated S-IgG responses throughout the study, maintaining a robust 100% response rate. A marginal decline occurred in only 4% of subjects at 14 days post-priming dose (Fig 2G). The S-IgM levels which were higher in this group than in the baseline S-IgG negative groups, showed a downward trajectory, and diminished two weeks after the priming dose (Fig 2H). Conversely, modest S-IgA responses persisted in most of these subjects, reaffirming the resilience of their immune profiles (Fig 2I). Datasets for this analysis are summarised in S2 Table.

### Distinct Spike-directed IgG and moderate IgA antibody response patterns in infection-naive and previously infected individuals with varied S-IgG serostatus

**Fig 3:**
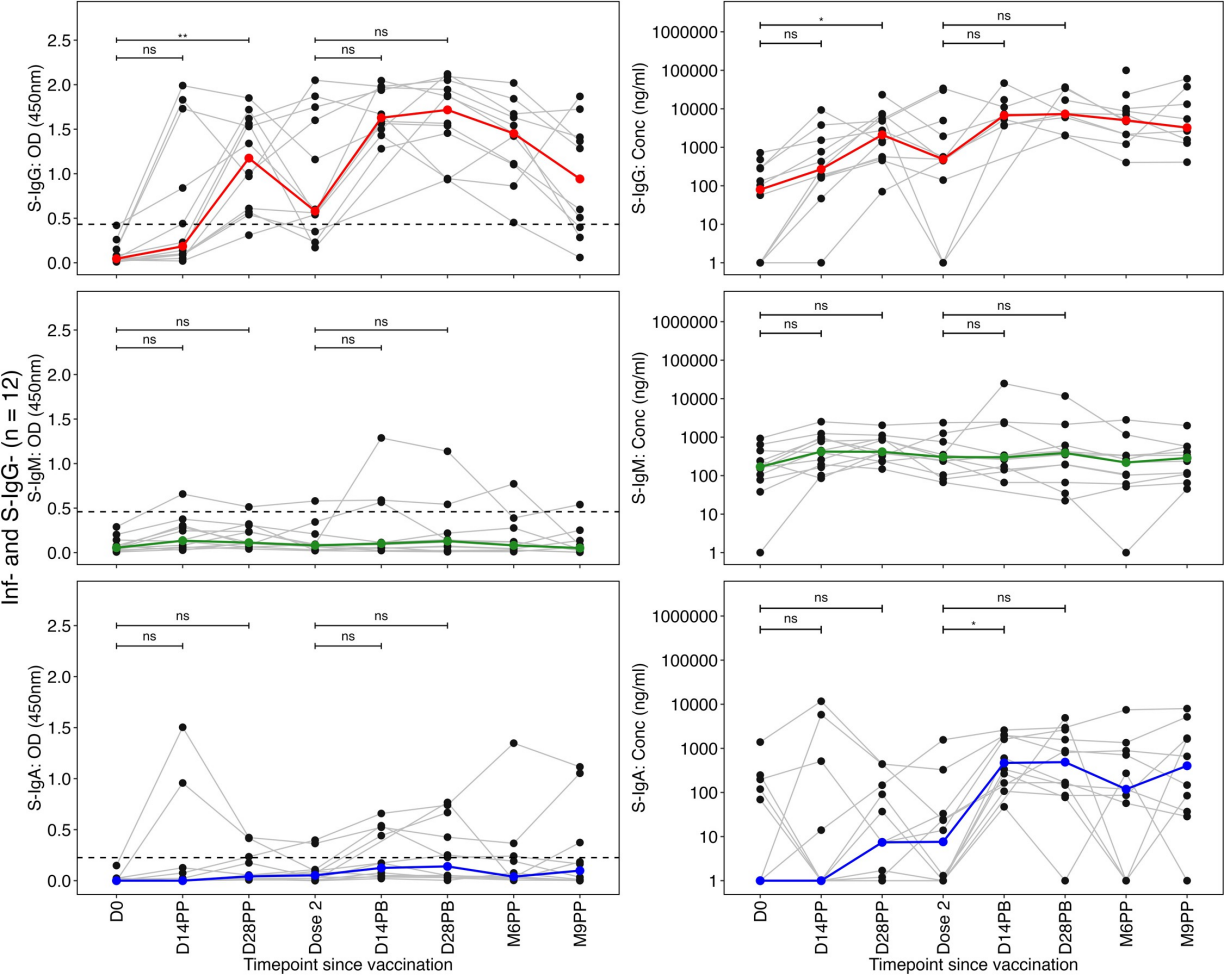
Individual profile plots of infection-naive participants who tested seronegative for S-IgG antibodies at baseline (Inf- and S-IgG-). **Fig 3** shows the individual profile plots (as grey lines) representing infection-naive participants who concurrently tested negative for S-IgG at baseline (Inf- and S-IgG-). In panels 3A-3F, the spike-directed antibody OD values and concentrations for S-IgG (Fig 3A and 3B), S-IgM (3C and 3D) and S-IgA (Fig 3E and 3F) are displayed throughout the study. The median antibody responses are depicted as solid-coloured lines, while the horizontal dotted lines indicate the OD cut-off values for S-IgG (0.432), S-IgM (0.459), and S-IgA (0.226). We used the unpaired Wilcoxon test to assess differences in antibody responses post-vaccination. The significance levels were denoted as not significant (ns) for p > 0.05, * for p ≤ 0.05, ** for p < 0.01, *** for p < 0.001, and **** for p < 0.0001.

In infection-naive individuals with no baseline S-IgG antibodies, a progressive increase in S-IgG antibody levels was noted from Day 0 to Day 14, culminating in peak levels on Day 28 following the initial dose. Before administering the booster dose, S-IgG levels exhibited an initial decline. Subsequently, there was a non-significant increase in these levels, reaching their peak on Day 28 post-boost. Following this peak, a gradual decline in S-IgG concentrations was observed (Fig 3A and 3B). Throughout the study, the levels of S-IgM and S-IgA antibodies exhibited sustained low responses, with only a marginal and statistically insignificant elevation in S-IgA antibodies observed following the booster.

**Fig 4:**
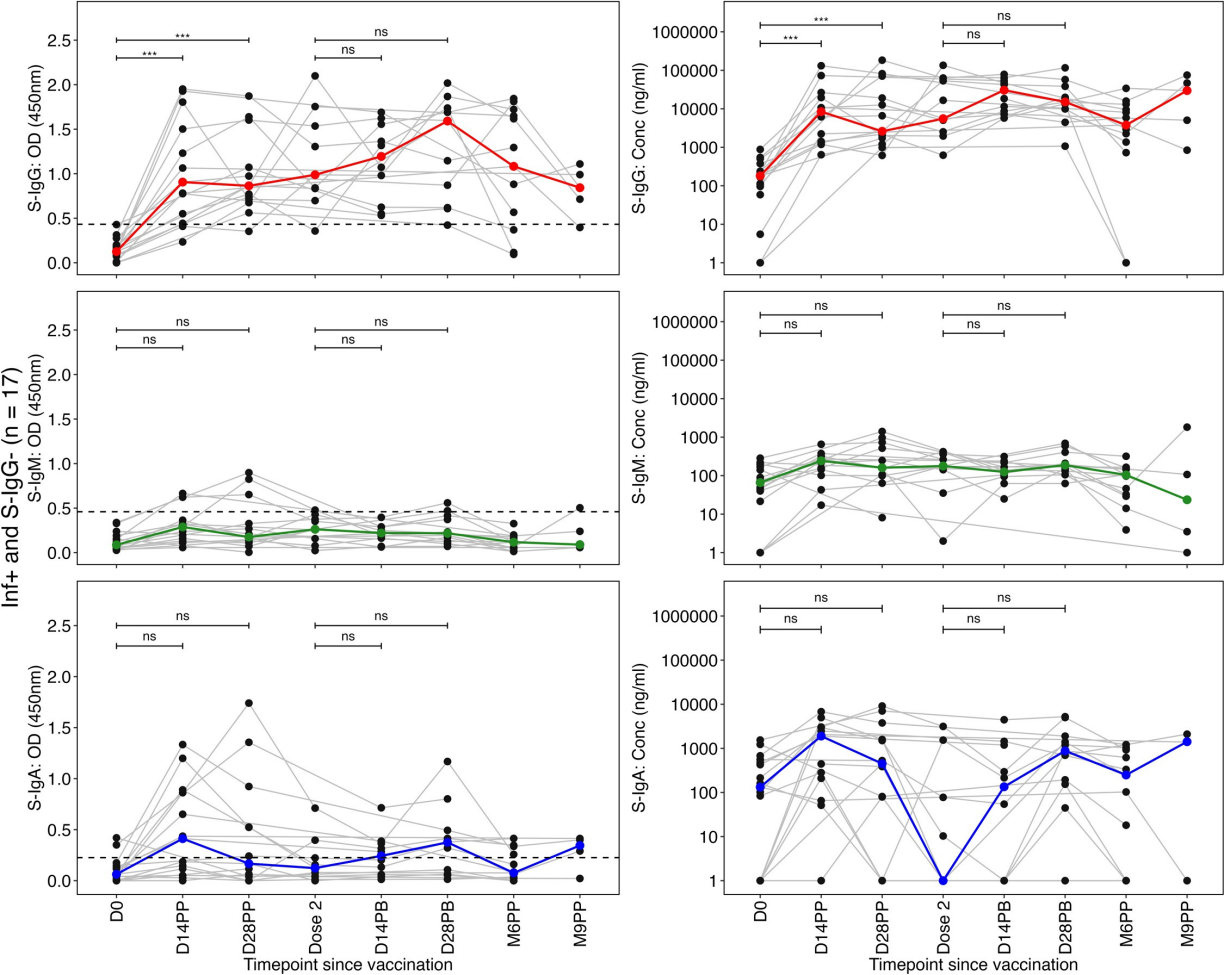
The distinct individual profile plots of participants who were initially infected but lacked baseline S-IgG seropositivity (Inf+ and S-IgG-). **Fig 4** illustrates the dynamic individual profile plots (grey lines) for participants initially infected but seronegative for Spike-directed IgG antibodies (Inf+ and S-IgG-) at baseline. The profiles (Fig 4A-4F) present the trajectory of their Spike-directed antibody responses over the study duration, including Spike-directed S-IgG (Fig 4A and 4B), S-IgM (Fig 4C and 4D), and S-IgA (Fig 4E and 4F). Bold coloured lines represent the medians of the antibody responses, while dashed horizontal lines indicate the reference OD cut-off values: S-IgG at 0.432, S-IgM at 0.459, and S-IgA at 0.226. We used the unpaired Wilcoxon test to assess differences in antibody responses post-vaccination. The significance levels were denoted as not significant (ns) for p > 0.05, * for p ≤ 0.05, ** for p < 0.01, *** for p < 0.001, and **** for p < 0.0001.

Among individuals who previously tested positive for the infection (Inf+) but lacked detectable S-IgG antibodies at the vaccination baseline (S-IgG-), a significant rise in S-IgG antibody levels occurred at 14 and 28 days post prime compared to their vaccination baseline levels at D0, illustrated in Fig 4A. Following the administration of a booster dose, no statistically significant increase in S-IgG antibody levels was observed. Instead, antibody concentrations showed a gradual rise, reaching their peak 14 days post-boost. However, in the subsequent months, there was a decline in antibody levels, eventually returning to near-baseline values six months after the initial vaccination. Notably, a significant ten-log-fold increase in antibody concentrations was observed between the sixth and ninth months, suggesting potential reinfection events, as shown in Fig 4B.

Both the primary and booster vaccine doses did not elicit a substantial increase in the levels of S-IgM (Fig 4C and 4D) and S-IgA (Fig 4E and 4F).

**Fig 5:**
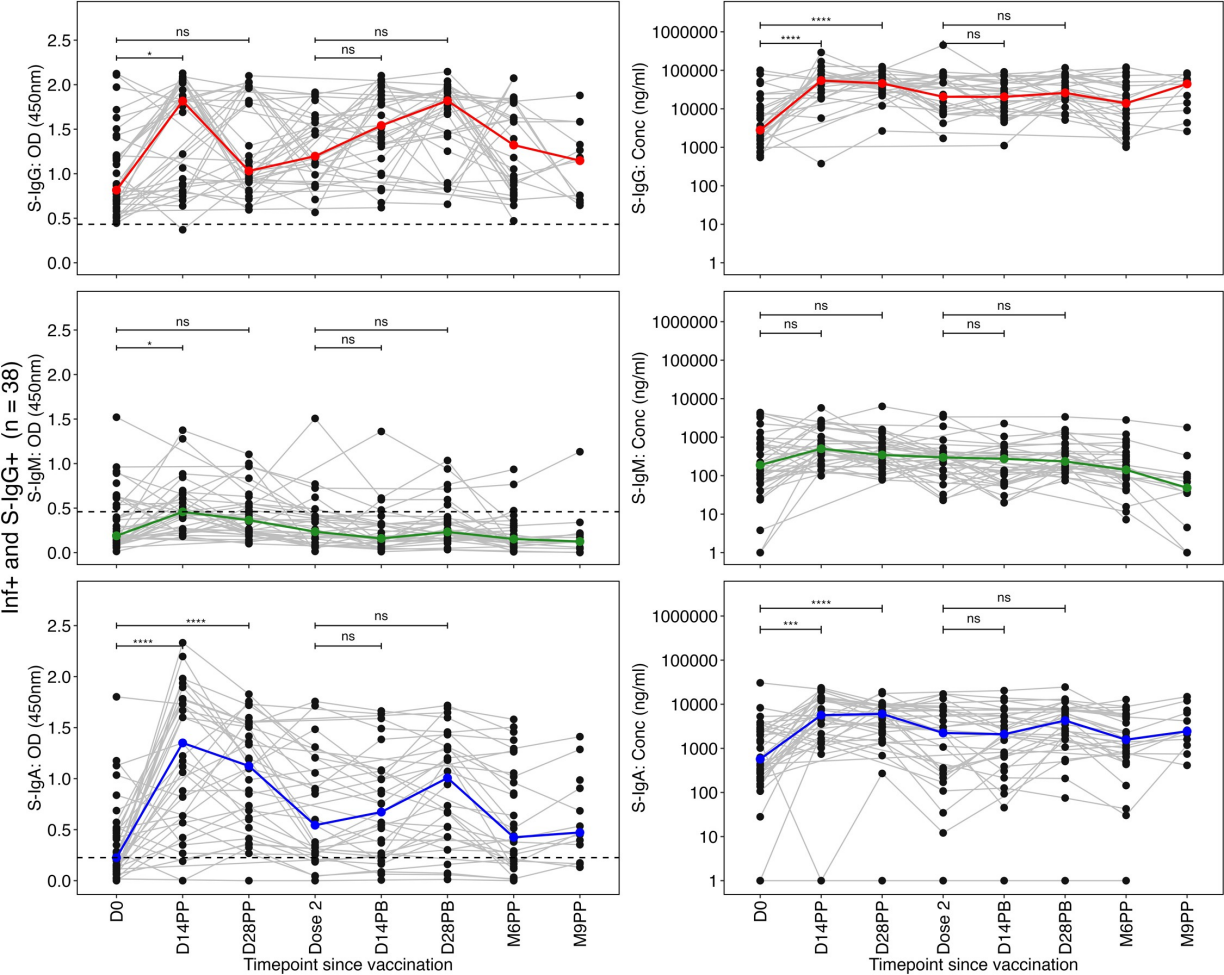
Dynamic trajectory of spike-directed antibody responses in previously infected baseline seropositive individuals (Inf+ and S-IgG+). **Fig 5** shows individual profile plots (grey lines) delineating participants initially infected and seropositive for Spike-directed IgG antibodies (Inf+ and S-IgG+) at baseline. These profiles (Fig 5A–5F) delineate the trajectory of their Spike-directed antibody responses across the study duration, encompassing Spike-directed S-IgG (Fig 5A and 5B), S-IgM (Fig 5C and 5D), and S-IgA (Fig 5E and 5F). The boldly coloured lines denote median antibody responses, while dashed horizontal lines denote reference OD cut-off values: S-IgG at 0.432, S-IgM at 0.459, and S-IgA at 0.226." We used the unpaired Wilcoxon test to assess differences in antibody responses post-vaccination. The significance levels were denoted as not significant (ns) for p > 0.05, * for p ≤ 0.05, ** for p < 0.01, *** for p < 0.001, and **** for p < 0.0001.

In previously infected individuals with baseline S-IgG antibody seropositivity, S-IgG antibody OD levels significantly rose by day 14 following the initial vaccination but declined by day 28 after the priming dose. Thereafter, the OD levels rose slightly and remained relatively stable subsequently (Fig 5A). The S-IgG antibody concentrations rose significantly at 14 and 28 days relative to the first dose, and maintained this elevation post-boost, followed by a noticeable increase six months after the primary dose (Fig 5B). S-IgM antibody levels were consistently low throughout the study period (Fig 5C and 5D). S-IgA levels peaked rapidly after the priming dose, peaked at 14 and 28 days, then declined but stayed marginally stable after the booster dose (Fig 5E and 5F).

### Differential dynamics of SARS-CoV-2 antibody concentrations from immunologically naive and previously infected cohorts

**Fig 6:**
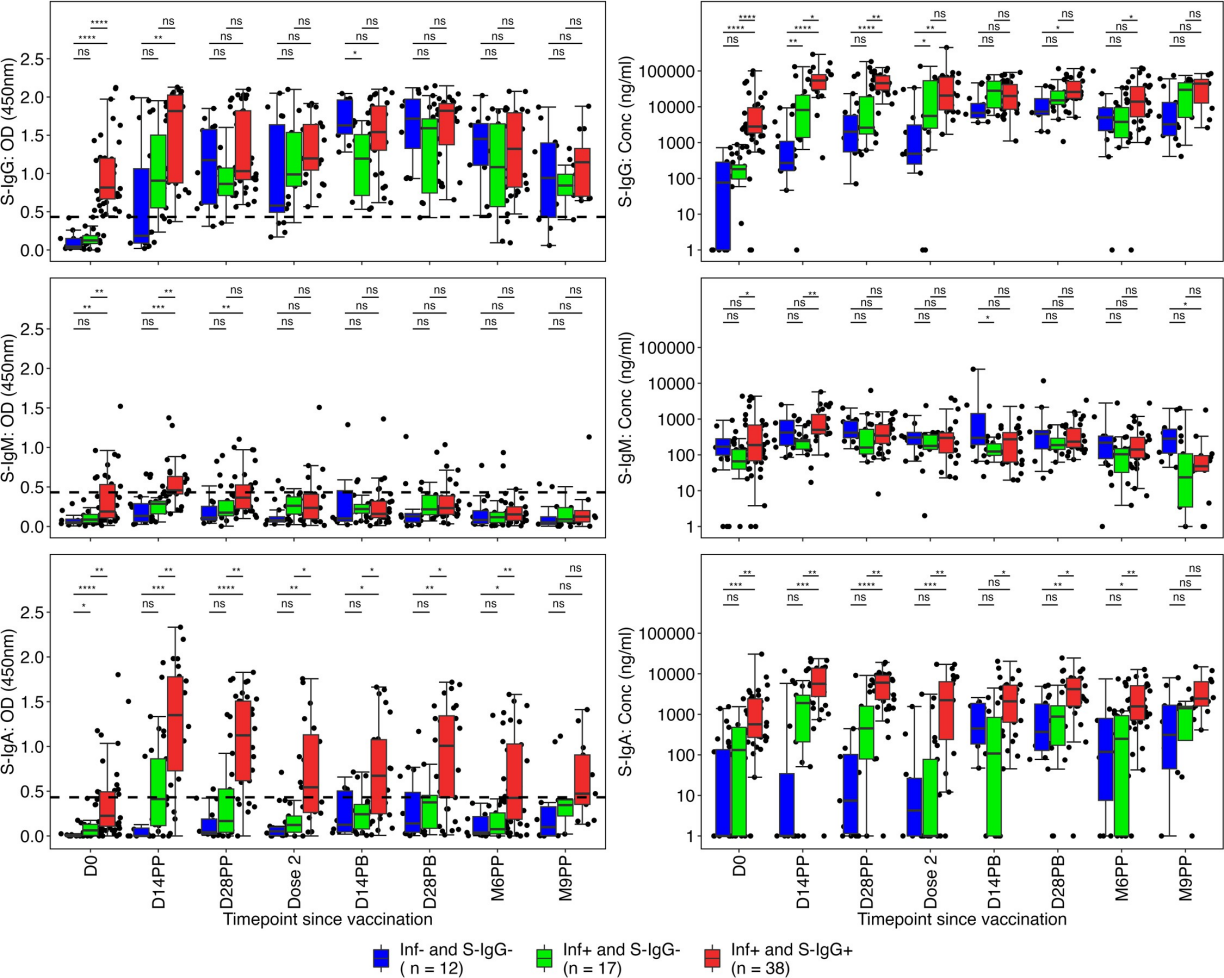
Box plots to compare spike-directed antibody responses across the three subgroups over time. **Fig 6** illustrates the box plots that compare the spike-directed S-IgG (Fig 6A and 6B), S-IgM (Fig 6C and 6D), and S-IgM (Fig 6E and 6F) antibody responses across the three subgroups over time. The statistical analysis employed an unpaired Wilcoxon test with Bonferroni correction for multiple testing, with p-values less than 0.05 indicating significance. Significance levels are denoted as follows: ns (non-significant) for p > 0.05, * for p ≤ 0.05, ** for p < 0.01, *** for p < 0.001, and **** for p < 0.0001.

After the initial dose, participant S-IgG seropositivity at baseline showed median S-IgG antibody OD levels that improved over two weeks but remained below optimal thresholds. Subsequently, there was an upward trend in antibody levels, eventually aligning with those observed in cohorts with prior infection, persisting at all subsequent time points. At the 14-day mark after the booster dose administration, the S-IgG OD levels matched and surpassed those of the baseline S-IgG seropositive group, as shown in Fig 6A.

The comparison consistently showed lower S-IgG levels in the naive group compared to the previously infected cohorts. This difference remained until the booster dose was administered, with the intriguing observation that the booster dose did not significantly elevate the antibody concentrations in the previously exposed individuals. The absence of significant differences in the elicited S-IgG concentrations across the three groups during the 14-day post-boost assessment suggested that antibody levels remained stable. However, a noticeable increase in antibody concentrations was observed at the 9-month mark, potentially indicating the emergence of reinfection. At nine months, the concentrations of induced S-IgG antibodies showed no significant differences among the three groups, as illustrated in Fig 6B.

Median S-IgM antibody OD levels remained consistently below the cut-off in all groups throughout the study, except for a significant spike observed at the 14-day post-priming evaluation in the cohort with prior exposure, surpassing the established cut-off, as shown in Fig 6C. Corresponding concentrations were consistently lower in the naive group, except at the 14-day post-boost time point, as illustrated in Fig 6D. The S-IgA OD levels and concentrations were consistently higher within the previously infected cohort for up to 6 months after the initial priming dose, as illustrated in Fig 6E and 6F, underscoring the benefits of herd immunity.

## Discussion

In the relentless battle against SARS-CoV-2, it is crucial to comprehensively understand the timeline of vaccine-driven immune responses, especially in populations with varying prior SARS-CoV-2 infections and baseline S-IgG antibody exposure histories. This study expounded on the post-ChAdOx1-S, Covishield vaccination antibody dynamics among three distinct subsets: individuals without prior infection and with a baseline of S-IgG negativity (Inf- and S-IgG-), those with prior infection and S-IgG negativity at baseline (Inf+ and S-IgG-), and those with prior infection and a baseline of S-IgG positivity (Inf+ and S-IgG+). Our goal was to offer insights that could inform vaccination protocols and enhance our understanding of vaccine-induced immune responses in real-world scenarios. Analysis of 67 subjects, segregated into three discrete exposure histories, yielded insights into the sequential and qualitative changes, revealing unique patterns influenced by prior infection history and the baseline S-IgG serostatus. In the cohort lacking prior infection, significant S-IgG seroconversion was seen, particularly after the first dose, accompanied by limited change in S-IgM and S-IgA OD levels. In subjects previously infected but S-IgG-negative at baseline, a gradual rise in S-IgG responses was observed, with simultaneous S-IgM decline and enduring S-IgA presence. In contrast, individuals previously infected and baseline S-IgG-positive showed high S-IgG levels and varying S-IgM and S-IgA dynamics. These data confirm the potency of hybrid immunity antibody kinetics following vaccination of the prior exposed. Importantly, these subgroups revealed the dynamic interplay of S-IgG, S-IgM, and S-IgA antibodies, highlighting the multifaceted nature of the antibody responses elicited by the ChAdOx1-S, Covishield vaccine.

Across the three cohorts, intriguing kinetics were revealed, with S-IgG antibodies emerging as the most pivotal due to their sustained and robust responses. The differential antibody dynamics observed in the three subgroups had implications for long-term immunity and vaccine efficacy. The findings suggest that prior infection and baseline S-IgG positivity provided a robust foundation for sustained and potent antibody responses. In contrast, immunologically naive individuals exhibited the ability to generate rapid and robust antibody responses following vaccination, which could align with or even surpass those seen in previously infected individuals. Robust S-IgG maintenance in previously infected individuals, regardless of baseline S-IgG status, signifies a sustained shield against reinfection. These observations suggest the ChAdOx1-S, Covishield vaccine’s effectiveness across diverse immune backgrounds, with implications for long-term protection. However, the data also illuminated distinct patterns within S-IgM and S-IgA responses, suggesting their distinct roles in the evolving immune landscape. The varying S-IgM and S-IgA kinetics highlight the complexity of immune memory, suggesting considerations based on infection history in cases of challenges with vaccine doses. Furthermore, S-IgA exhibited a blend of persistence and variability, characterised by elevated levels in individuals with prior infection, suggesting good signaling of memory B-cell activation. The sustained S-IgA presence in certain groups augments the concept of the contribution of mucosal immunity to protection. These outcomes emphasise that a one-size-fits-all approach may not be optimal, and personalised vaccination strategies might enhance immune outcomes. The rapid seroconversion in infection-naive individuals aligns with prior studies [17, 20, 21], highlighting the vaccine’s potential for eliciting robust antibody responses. The sustained S-IgG levels in previously infected subjects and varying S-IgM and S-IgA trends indicate a differential memory response that hinges on prior exposure [9, 18]. The substantial differences in S-IgA concentrations across subgroups emphasised the role of mucosal immunity in shaping memory responses [22, 23].

These data support existing literature and offer intricate insights into the interplay between immune history and vaccine-induced responses. The findings align with prior research on the dual role of S-IgG antibodies, serving as both markers of past exposure [7, 17] and mediators of immune memory [7, 24]. The persistent S-IgG response in the previously infected highlights the potential for prolonged immunity. In contrast, the notable surge in S-IgG levels among immunologically naive individuals align with the vaccine’s potential for inducing rapid and potent primary antibody responses in this population [7, 17]. The unique dynamics observed in S-IgM and S-IgA profiles hint at intricate interactions among various immune components, warranting further in-depth exploration. These observations align with studies that emphasise the synergy among immune components in shaping effective protective immunity [25, 26]. Robust S-IgG maintenance in previously infected individuals could result from memory B cell activation and differentiation [27], while the S-IgM decline may signify a shift towards class-switched antibody production [28, 29]. The sustained S-IgA response could reflect local immune memory within mucosal tissues, aligning with higher levels of IgG and IgA antibodies in convalescents due to convergent antibody response [28, 30]. Vaccine-induced immune responses in previously infected individuals are likely boosted by an anamnestic response, explaining the rapid and robust S-IgG surge post-vaccination [21, 31, 32].

This study’s implications extend to vaccine policy and immune response prediction. Incorporating prior infection history and baseline S-IgG status into vaccine strategies might optimise antibody responses and durability. The data also hint at a possible predictive value of early post-vaccination responses in naive individuals, aiding in estimating eventual immunity. These insights are pivotal in refining global vaccination campaigns and reinforcing our comprehension of SARS-CoV-2 immune dynamics.

While this study presents compelling insights, certain limitations merit consideration to provide a more comprehensive understanding and holistic approach. The study’s limitations include a relatively modest sample size and a focus on antibody responses, which constitute one facet of the immune response. The sample size might benefit from expansion for broader generalization. Exploring cellular immune responses could enrich our understanding of the comprehensive immune landscape post-vaccination. Additionally, longer follow-up periods would enable tracking of antibody kinetics beyond 9 months, providing more insights into the durability of responses over extended periods and during subsequent exposures. Finally, at the onset of the COVID-19 epidemic in Uganda, specimens were collected as part of a program aimed at detecting and isolating individuals who tested positive via PCR. Notably, HIV serostatus testing was not a criterion for inclusion in this study. Thus, our objective was to assess antibody responses in a real-life scenario, we did not stratify the cohort analysis by HIV status. However, we acknowledge this as a limitation of the study, given that previous research in this population has indicated lower antibody responses among HIV-positive individuals. Future investigations could encompass larger cohorts, diverse vaccine regimens, and T-cell responses to achieve a more comprehensive and holistic understanding.

In conclusion, our research demonstrated a relationship between previous infection, initial S-IgG antibody levels, and the immune responses triggered by the ChAdOx1-S, Covishield vaccine. The vaccine showed consistent immunogenicity across various immune backgrounds, offering insights for pandemic control strategies. This study examined the dynamics of S-IgG, S-IgM, and S-IgA antibodies, offering insights into COVID-19 immunity. The findings contributed to a better understanding of vaccination strategies, enhanced hybrid immunity, and guided management of the COVID-19 pandemic.

## Supporting information

S1 Table S1 Table presents the raw data on antibody responses, specifically IgG, IgM, and IgA, measured as optical densities at 450 nm and corresponding concentrations in ng/ml. The data includes measurements from various time points: baseline (D0), day 14 post-prime (D14 PP), day 28 post-prime (D28 PP), at the second dose, day 28 post-boost (D28 PB), six months post-prime (M6 PP), and nine months post-prime (M9 PP). Data is presented for IgG, IgM, and IgA directed against the spike (S) and nucleocapsid (N) proteins at each time point.
(XLSX)

S2 Table S2 Table presents the demographic data of the study participants, including age and gender. (XLSX)

S3 Table S3 Table presents a database detailing the percentage of individuals with IgG, IgM, and IgA antibodies directed against the spike (S) and nucleocapsid (N) proteins at each time point. (XLSX)
